# Bioassay-guided isolation of Fenghuang Dancong tea constituents with α-glucosidase inhibition activities

**DOI:** 10.3389/fnut.2022.1050614

**Published:** 2022-11-10

**Authors:** Hua Zhou, Jin Liao, Juanying Ou, Jiayu Lin, Jie Zheng, Yifei Li, Shiyi Ou, Fu Liu

**Affiliations:** ^1^Department of Food Science and Engineering, Jinan University, Guangzhou, China; ^2^Institute of Food Safety & Nutrition, Jinan University, Guangzhou, China

**Keywords:** flavonoids, bioassay-guided isolation, tea, α-glucosidase inhibition, antidiabetic compounds

## Abstract

An α-glucosidase inhibition assay showed the ethanolic extract of Fenghuang Dancong tea had potential α-glucosidase inhibitory activity. The most bioactive fraction, which was obtained *via* bioassay-guided isolation of the extract, was further purified to create five compounds, including three novel compounds (**1**–**3**). These compounds were analyzed and identified in detail using high-resolution-mass spectrometry and extensive one-dimensional and two-dimensional NMR spectroscopy experiments. Among the compounds, compound **1** contained *cis* double bonds and showed the strongest α-glucosidase inhibitory activity with an IC_50_ value of 7.51 μM, which is significantly lower than that of compound **2** with *trans* double bonds. Enzyme kinetic experiments showed that **1** was a reversible non-competitive α-glucosidase inhibitor.

## Introduction

Diabetes (DM) is a common disease characterized by hyperglycemia. It can be divided into type 1 and type 2 diabetes based on insulin dependence (1). Currently, there are about 451 million people with diabetes in the world. It is expected that with the improvement of living standards in the future, the number of people suffering from diabetes may increase significantly (2). The pathogenesis of type 2 diabetes (DM) mainly results from the failure of insulin to correctly regulate carbohydrate metabolism, which leads to increased blood sugar (3). α-Glucosidase, an enzyme present in the small intestine, plays an important role in blood glucose levels. Inhibiting α-glycosidase activity contributes to the reduction of blood glucose (4). Acarbose, miglitol, and daunorubicin are α-glucosidase inhibitors commonly used in diabetic patients, but these drugs have many side effects (5). Therefore, discovering new plant-derived drugs to treat DM would be beneficial.

Recent research on preventing diabetes has focused on natural products, especially from edible plants, because these substances are relatively safe and low-cost (3). Types of tea manufactured with *Camellia sinensis* L as the raw material are popular beverages in East Asia (6). Although there are significant differences in the chemical compositions of different types of tea, flavonoids are the major components. Our previous studies investigated the constituents of Yingde black tea, including quercetin, (–)-epicatechin (EC), apigenin, (–)-gallocatechin gallate (GCG), and amentoflavone, and amelliaone **A** exhibited α-glucosidase inhibitory activity with an IC_50_ value of 10.2 μM, which was lower than that of acarbose (7). This result implied potential anti-diabetic activity of tea.

Fenghuang Dancong tea, such as Yingde black tea, is made from the leaves of *Camellia sinensis* L. However, there are significant differences in the processing methods between the two types of tea. Fenghuang is a semi-fermented tea (8) and Yingde is a post-fermented tea (9). Fenghuang Dancong tea is desired by consumers because of its characteristic flavor and taste, and until now, the nonvolatile components of Fenghuang Dancong tea have been seldom reported (10). The chemical composition, flavor, aroma, and nutritional functionality of tea are closely related to the physical and climatic environment, production technology, and other factors. Based on the reasons described above, we decided to choose Fenghuang Dancong tea for this research. With the aim of identifying natural antidiabetic compounds from tea, the bioassay-guided isolation of α-glucosidase inhibitors from Fenghuang Dancong tea was performed.

## Materials and methods

### General

Phosphate buffer saline (PBS), acarbose, metformin (Met), fetal bovine serum (FBS), penicillin (PEM), insulin, streptomycin (SM), and dimethyl sulfoxide (DMSO) were obtained from Aladdin (Shanghai, China). The compounds 4-nitro-phenyl-α-d-gluco-pyranoside (p-NPG), 2,4,6-tris- (2-pyridyl)-*s*-triazine, and α-glucosidase, were purchased from J&K Scientific (Beijing, China). The HepG2 cell lines and a glucose kit were bought from the American Type Culture Collection (ATCC, Rockville, MD, USA) and Nanjing Jiancheng Biological Engineering Research Institute (Nanjing, China). Fenghuang Dancong tea was purchased from Qiqun tea corporation (Chaozhou, China). Thin layer chromatography was prepared by using silica gel 200 F_254_ (Haiyang Co., Qingdao, China). High-performance liquid chromatography (HPLC) was an Agilent 1260 (Agilent, Palo Alto, CA, USA) equipped with UV detector and a YMC-Pack-C18 column (250 mm × 10 mm, 5 μm). Nuclear magnetic resonance spectra (NMR), IR spectra, UV-vis spectra, and high-resolution spectra (HR–ESI–MS) were recorded using an AVANCE NEO 400 MHz (Fällanden, Switzerland), a Fourier-transform-infrared-spectrophotometer (Thermo Electron Co., Waltham, MA, USA), a 53 UV-vis spectrophotometer (Lengguang Technology Co., China), and a Bruker maXis mass spectrometer (Fällanden, Switzerland), respectively.

### Extraction and separation

Approximately 2.0 kg of Fenghuang Dancong tea was extracted three times with 20 L of 95% ethanol and manually stirred according to a previously published method (7). The crude extract (120.4 g) was obtained by removing ethanol under reduced pressure and then suspending in water. The crude extract was sequentially extracted with three solvents (first dichloromethane, then ethyl acetate, and finally n-butanol). After evaporation of the solvent, the yield of the dichloromethane partition (AD) was 15.5 g; the ethyl acetate partition (AE) was 16.8 g; the n-butanol partition (AN) was 20.3 g; and the residual water partition (AR) was 29.8 g.

AE fractions (ethyl acetate partition, 16.8 g) were separated by column chromatography on silica gel (400 g), and dichloromethane, ethyl acetate, and methanol were used as the eluents for gradient elution as follows: 100% dichloromethane for 40 min, followed by gradually increasing the proportion of ethyl acetate to 100% over 120 min, and eluting continuously for 40 min under this condition. Finally, the proportion of methanol was slowly increased to 100% over 120 min, and the sample was eluted continuously for 30 min. TLC was used to monitor the elution process, with dichloromethane/methanol (1:10, v/v) as the developing agent. According to the TLC results, the tube of the collected AE fraction was divided into six subfractions, i.e., subfractions 1–6. Then, the glucosidase inhibitory activity of each subfraction was evaluated. It was found that subfraction 3 had the highest activity, and it was subjected to further separation and purification. Fraction 3 showed only one spot on the TLC plate, but two chromatographic peaks were observed by HPLC [the mobile phase: MeOH/H_2_O (1:10, v/v)]. Thus, preparative HPLC was employed to obtain compounds **1** (10 mg) and **2** (15 mg). Fraction 3B was first separated with a Sephadex LH-20 column [the eluent: MeOH/H_2_O (1:1, v/v)]. Similar fractions were integrated and monitored by TLC, using petroleum ether/ethyl acetate (1:10 v/v) as the developing agent. Finally, preparative HPLC was used for purification to obtain compounds **4** (25.0 mg, *t*_R_ =19.2 min). Fraction 3C was first separated on a 20 g silica gel column using dichloromethane/acetone (1:1, v/v) as the eluent and then purified on a RP_18_ column using a gradient of acetone and water (from 10:1 to 1:10) for elution. Finally, compound **2** (5.6 mg) was isolated by preparative TLC. Fraction 4D was first separated on a Sephadex LH-20 column, using 100% MeOH, and then purified on a RP_18_ (200 g) column using 70% methanol. Finally, compound **3** (8.8 mg, R*f* = 0.5) was obtained *via* preparative TLC.

### α-glucosidase inhibition assay

This experiment was carried out according to the literature (11). Briefly, the test sample was dissolved in solutions (SS) with DMSO as the solvent. p-NPG and α-glucosidase were, respectively mixed with phosphate buffer (PB, 60 mM phosphate, pH 6.8) and used as stock solutions. The concentration of α-glucosidase solution (GS) was 0.5 U/ml and that of p-NPG solution (NS) was 5 mM. In addition, four different solutions (A, B, C and D) were prepared: Solution A contained 112 μl PB, 20 μl GS, and 8 μl SS; Solution B contained 112 μl PB and 20 μl GS; Solution C contained 112 μl PB, 8 μl SS, and 20 μl GS; and Solution D contained 132 μl PB and 8 μl DMSO. After starting each reaction at 37°C, *p-*nitrophenol was quantified by measuring the absorbance at 405 nm with a UV–vis spectrophotometer. The inhibition rate was calculated according to equation (1):


(1) $$\begin{array}{l} \text{Inhibition rate }(\%)\\ =\;[((A_{as}\;-\;B_{as})(C_{as}\;-\;D_{as}))\;/\;(A_{as}\;-B_{as})]/100, \end{array}$$

where A_as_, B_as_, C_as_ and D_as_ are the absorbances of solution A, B, C and D, respectively. The sample concentration that inhibited 50% α-glucosidase activity was defined as the IC_50value._

### Glucose consumption assay

Here, HepG2 cells were used to evaluate the glucose consumption of the isolated compounds according to a method described in the literature (12). Briefly, HepG2 cells were cultured for 24 h. Subsequently, they were transferred from DMEM medium (containing FBS, PEM and SM) to another DMEM medium (containing 12.0 mM glucose). Then, HepG2 cells and the test samples with a concentration of 0.18 mg/L were incubated together for another 24 h. The glucose consumption was obtained by measuring the glucose content of the cell culture supernatant with a glucose kit.

## Results and discussion

### Bioactivity-guided fraction

The ethanol extract of Fenghuang Dancong tea had good anti-diabetes activity, as determined through a preliminary α-glucosidase inhibition assay. In order to separate and identify the most active chemical components, the ethanol extract was subjected to a segmentation process. The ethanol extract was suspended in water and extracted with three solvents of different polarity to obtain the corresponding fractions (AD, AE, AN, and AR, see section “Extraction and separation” for details). In addition, the concentration of the sample had an important impact on the evaluation of its biological activity, and the results obtained by different concentrations were not consistent. After repeated experiments, a concentration of 100 mg/ml was found to better distinguish the activity of different fractions.

**Figure 1:**
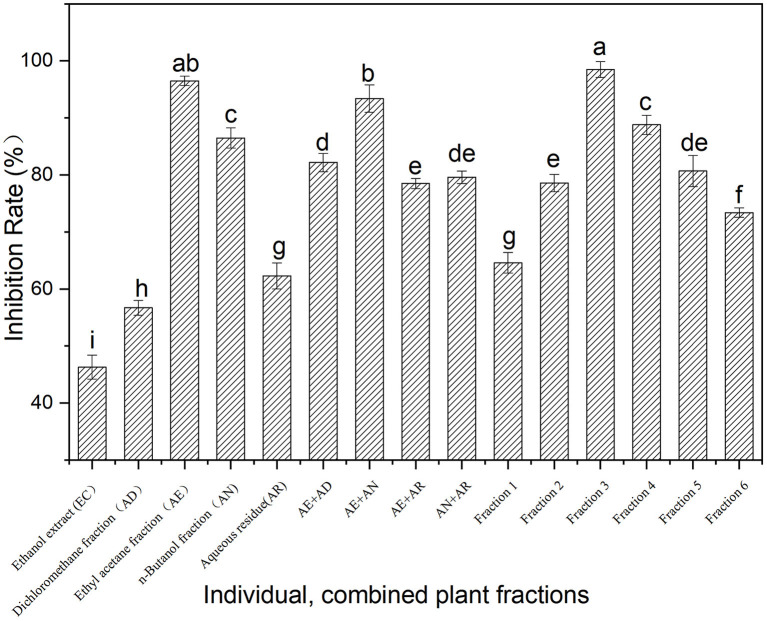
Inhibition on α-glucosidase activity of the fractions from Fenghuang tea. The values with different small letters (a–i) indicate a significant difference between different groups at *p* < 0.05.

The inhibition of the ethanol extract of Fenghuang Dancong tea and its fractions (AD, AE, AN, and AR) on α-glucosidase is shown in Figure 1. At the same concentration (100 mg/ml), the α-glucosidase inhibitory activity of the fractions (AD, AE, AN, and AR) was generally higher than that of the ethanol extract of Fenghuang Dancong tea (the combination of the four fractions). Among them, the ethyl acetate soluble fractions (AE) exhibited the highest inhibition rate of α-glucosidase (96.5%), followed by n-butanol soluble fractions and water-soluble fractions (AD). In order to clarify the synergistic effect of the fractions, the α-glucosidase inhibitory activity of the combined fractions was investigated. The results show the inhibitory activity of the combination of AE and fractions (AE + AN, 100 mg/ml) on α-glucosidase (93.4%) was generally lower than that of the individual fraction, i.e., the AE fraction (96.5%). Other combinations also had similar results. Thus, the moderate polarity of the fraction was conducive to improving the inhibitory activity. The fraction AE (ethyl acetate partition) with the highest inhibition rate was further isolated to create fractions 1–6 with an α-glucosidase inhibition rate ranging from 64.6% to 98.5% (Figure 1). Among fractions 1–6, fraction 3 showed the strongest α-glucosidase inhibitory activity, with an inhibition rate of 98.5%.

### Isolation of constituents from the bioactive fraction

**Figure 2:**
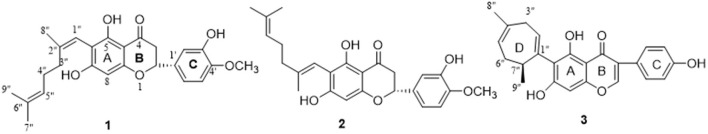
Structures of new compounds **1**–**3**.

The most bioactive fraction obtained *via* bioassay-guided isolation was further separated to afford five compounds, including three novel compounds. Compounds **4** (ameridone D) and **5** (ameridone E) are known compounds and have been isolated from Yingde black tea (7). Compounds **1**–**3** are new compounds (Figure 2), and they were analyzed and characterized in detail by a various spectroscopy techniques.

#### Spectroscopic data of three novel compounds 1–3

Amelliaone F(**1**): HR–ESI–MS (positive) *m/z* 425.1961; ${\left[\text{α}\right]}_{20}^{\text{D}}$-20.45 (c0.001, dichloromethane); IR(KBr) υ_max_: 3,448, 1,637, 1,458, 1,384, and 1,088 cm^−1^; UV (dichloromethane) λ_max_: 288 nm.

Amelliaone G(**2**): HR–ESI–MS (positive) *m/z* 425.1963; ${\left[\text{α}\right]}_{20}^{\text{D}}$-56.79 (c0.001, dichloromethane); IR(KBr) υ_max_: 3,443, 1,651, 1,384, and 1,083 cm^−1^; UV (dichloromethane) λ_max_: 286 nm.

Amelliaone H(**3**): HR–ESI–MS (positive) *m/z* 391.1542; ${\left[\text{α}\right]}_{20}^{\text{D}}$ + 78.23 (c 0.001, dichloromethane); IR(KBr) υ_max_: 3,677, 2,924, 1,635, 1,443, and 805 cm^−1^; UV (dichloromethane) λ_max_: 266 nm.

#### Identification of compounds 1–3

Compound **1** was a yellow solid with a melting point of 165°C, and it showed excellent liposolubility. From the infrared spectrum analysis, the peaks at 3,448, 1,637, 1,458, 1,384, and 1,088 cm^−1^ indicated that the molecule may contain a carbonyl group (1,637 cm^−1^) and a hydroxyl group (3,448 cm^−1^). In the UV spectrum analysis, a strong absorption peak appeared at 288 nm, indicating that the molecule may contain a benzene ring. High-resolution mass spectrometry (HR–ESI–MS) analysis showed that the peak of [M + H]^+^ was 425.1961, and the molecular formula was inferred to be C_25_H_28_O_6_ with unsaturation of 13. The ^1^H NMR (Table 1) analysis obtained a series of signals of functional groups in the molecule, including four aromatic protons (δH 6.09, 7.06, 6.89, and 6.90), which confirmed that the molecule contained a benzene ring, and a 2,7-dimethyl-1,5-diheptenyl group [5.82 (1H, H-1″), 1.93 (2H, H-2″), 2.08 (2H, H-4″), 5.09 (1H, H-5″), 2.02 (3H, H-3″), 1.52 (3H, H-8″), and 1.64 (3H, H-9″)], which was confirmed by heteronuclear multiple bond correlation (HMBC) analysis, as shown in Supplementary Figure S1. The characteristic signal of the dihydroflavonoid skeleton was observed in the ^13^C NMR spectrum (Table 1), including four aromatic methylene groups, seven aromatic quaternary carbons, one upfield methylene group (δC 79.0), one carbonyl group (δC 196.1), and one upfield methylene (δC 43.3). Based on the above information, compound **1** was preliminarily deduced to be a derivative of hesperetin (13). The position of the substituents (a 2,7-dimethyl-1,5-diheptenyl group) linked to hesperidin was determined by HMBC spectra. The correlation peaks [δH 5.82 (H-1″) with C-7 (δC 161.1), C-5 (δC 161.8), and C-6 (δC102.7)] in the HMBC spectrum indicated that a 2,7-dimethyl-1,5-diheptyl group was connected to the sixth carbon (C-6).

**Table 1:** ^1^H and ^13^C NMR data of **1**–**3** (**1**, **2** in CD_3_Cl and **3** in CD_3_OD).

**H/C**	**1**		**2**		**3**	
	**δH (mult., *J*)**	**δC**	**δH (mult., *J*)**	**δC**	**δH (mult., *J*)**	**δC**
2	5.34 (1H, m)	79.0	5.34 (1H, m)	79.2	8.12 (1H, s)	153.1
3	3.05, 2.77 (2H, m)	43.3	3.08, 2.78 (2H, m)	43.3		122.7
4		196.1		195.1		181.2
5		161.8		161.8		160.6
6		102.7		102.0		104.8
7		161.1		161.0		162.9
8	6.09 (1H, s)	94.5	6.10 (1H, s)	94.5	6.31 (1H, s)	98.4
9		161.5		161.1		153.5
10		105.8		105.9		104.1
1′		131.6		131.6		121.8
2′	7.06 (1H, s)	113.0	7.06 (1H, s)	113.0	7.41 (d,7.6 Hz)	130.0
3′		145.9		145.9	6.84 (d, 7.6 Hz)	114.1
4′		146.9		146.9		159.1
5′	6.89 (d, 9.0 Hz)	110.6	6.91 (d, 9.0 Hz)	110.6	7.41 (d, 7.6 Hz)	114.1
6′	6.90 (d, 9.0 Hz)		6.94 (d, 9.0 Hz)	118.2	6.84 (d, 7.6 Hz)	130.0
1^′′^	5.82 (1H, m)	113.6	5.81 (1H, m)	113.5		147.2
2^′′^		147.9		146.9	5.98 (1H, m)	109.7
3^′′^	1.93 (2H, m)	33.4	2.35 (2H, m)	39.1	2.17 (2H, m)	24.9
4^′′^	2.08 (2H, m)	26.6	2.31 (2H, m)	26.4		133.0
5^′′^	5.09 (1H, m)	123.0	5.17 (1H, m)	123.8	5.34 (1H, m)	126.7
6^′′^		132.6		133.2	2.03 (2H, m)	31.0
7^′′^	1.52 (3H, s)	17.5	1.67 (3H, s)	17.6	2.98 (1H, m)	38.3
8^′′^	2.02 (3H, s)	23.5	1.54 (3H, s)	17.7	1.68 (3H, s)	22.1
9^′′^	1.64 (3H, s)	25.8	1.73 (3H, s)	25.7	1.26 (3H, s)	21.2
–OCH_3_	3.97 (3H, s)	56.0	3.94 (3H, s)	56.3		

Another issue that should be clarified is the configuration of the double bond on the substituent. First, the *cis* and *trans* isomers of the compound were optimized using Gaussian 16 software to obtain the conformation with the lowest energy, as shown in Supplementary Figure S2. Then, the spatial distances of the two groups of key protons (H-7”/H-1” and H-1'/H-3”) were measured. It was found that the distance (2.24 Ȧ) between H-7″and H-1”in the compound with *cis* configuration was much shorter than that of the compound with *trans* configuration (3.76 Ȧ). In the ROESY spectrum of compound **1**, a ROESY correlation of H-7^′′^(δ2.02)/ H-1^′′^(δ5.82) indicated that the substituent of the compound **1** contained a *cis* double bond. In addition, the compound contained a chiral center, and its conformation was obtained by comparing the optical rotation value with the parent compound hesperidin. The optical rotation of the compound **1** was negative and the same as that of hesperidin; therefore, the configuration of C-3 was assigned as *R*. In conclusion, compound **1** was characterized as (*R, z*)-6-(2,6-xylheptane-1,5-dien-1-yl)-5,7-dihydroxy-2-(3-hydroxy-4-methoxyphenyl) chroman-4-one, which is a novel C-pentenylated flavanone named Amelliaone F.

Compound **2** had almost the same melting point (165°C) and liposolubility as compound **1**, which was also a yellow solid. The HR–ESI–MS spectrum showed that the [M + H ]^+^ peak of the compound was 425.1963, and its molecular formula was deduced to be C_25_H_28_O6, which was the same as that of compound **1**. In addition, the NMR spectra (Table 1), HR, and UV spectra showed the two compounds had great similarities. However, it can be seen from HPLC that the two compounds have different retention times. There were also subtle differences in the ^13^C NMR spectra: two methylene signals on the NMR spectrum of compound **2** (δ39.1 and δ17.7) were significantly different from that of compound **1**. It was preliminarily inferred that these two were isomers, and this hypothesis was confirmed by HMBC spectrum. To further validate this, ROESY was used again to determine the configuration of the double bond in this compound. H-3″ and H-1″ had ROESY correlations for compound **2**, and H-7″ and H-2″ had correlations for compound **1**. This clearly indicates that the two compounds were *cis-tran*s isomers of each other and that the configuration of a double bond (C1^′′^/C4^′′^) in compound **2** was *trans*. Thus, compound **2** was determined as (R,E)-6- (2,6-dimethylhepta−1,5- dien-1-yl)-5,7-dihydroxy-2- (3-hydroxy-4 -methoxyphenyl) chroman- 4-one, which is a C-prenylated flavanone named Amelliaone G.

Compound **3** was a yellow solid, similar to compounds **1** and **2**, but its liposolubility was slightly inferior to the others. The compound was analyzed by the HR–ESI–MS to obtain a [M + H]^+^ peak of 391.1542, and its chemical formula was determined to be C_24_H_22_O_5_. We preliminarily deduced that the compound contained a parent isoflavone from the infrared, ultraviolet, and NMR spectra (Table 1). In the ^13^C-NMR spectrum, the remaining nine carbon signals, except for the signals of the parent isoflavone, formed a seven-membered ring substituted by dimethyl and assigned as D-ring (consisting of three methyne groups, two quaternary carbon atoms, two methene groups, two methyl groups). This result was further confirmed by the correlation peaks (H-3^′′^/C-1^′′^, C-5^′′^; H-8^′′^/C-5^′′^, C-4^′′^; H-9^′′^/ C-6^′′^,C-1^′′^) in the HMBC spectrum. The position of the D-ring connected to the parent isoflavone was easily resolved by the HMBC spectrum. Correlation peaks between H-2^′′^/C-6 and H-7^′′^/C-6^′′^ were clearly identified and indicated the D ring was attached at the sixth carbon (C-6). Compound **2** had one chiral center, and its conformation was determined by the positive or negative value of its optical rotation. Different from the optical rotation values of compounds **1** and **2**, that of compound **3** was positive, and the conformation of compound **3** was determined to be *S*. The resulting structure of compound **3** was identified as (S)-6-(4,7-dimethylcyclohepta-1,4-dien-1-yl)- 5,7- dihydroxy-3-(4-hydroxyphenyl)−4H- chromen-4-one, based on spectral evidence. Thus, compound **3** was identified as Amelliaone H.

**Figure 3:**
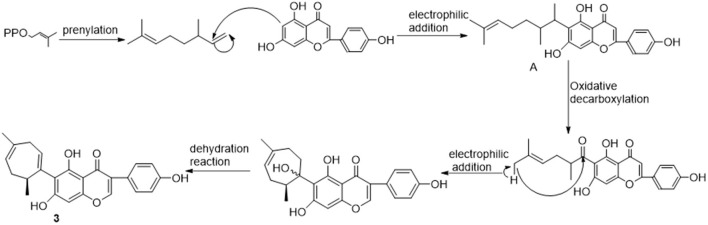
Hypothetical biosynthetic pathways of compound **3**.

The above three compounds were all prenylated flavonoids. The distribution of such compounds in nature is common, and they generally have strong biological activities. The biosynthesis of prenylated flavonoids is generally considered to include a series of reactions with flavonoids as the substrate, such as crenulation, as well as oxidation and reduction reactions. The formation or transformation of these compounds is easily influenced by genotype, sunshine duration, air humidity, ambient temperature, and processing methods (14), which is similar to the transformation of catechin in tea (15). Here, compound **3** was taken as an example to briefly deduce the biosynthetic process (Figure 3). First, the substrate isoflavone was subjected to prenylation and electrophilic addition reaction to form an intermediate A, followed by oxidative decarboxylation, electrophilic addition, and dehydration reaction to form the final product. The effects of tea processing on these compounds in Fenghuang Dancong tea are worthy of in depth and systematic research.

### α-glucosidase inhibitory activity

#### Effect of compounds on α-glucosidase inhibitory activity

**Figure 4:**
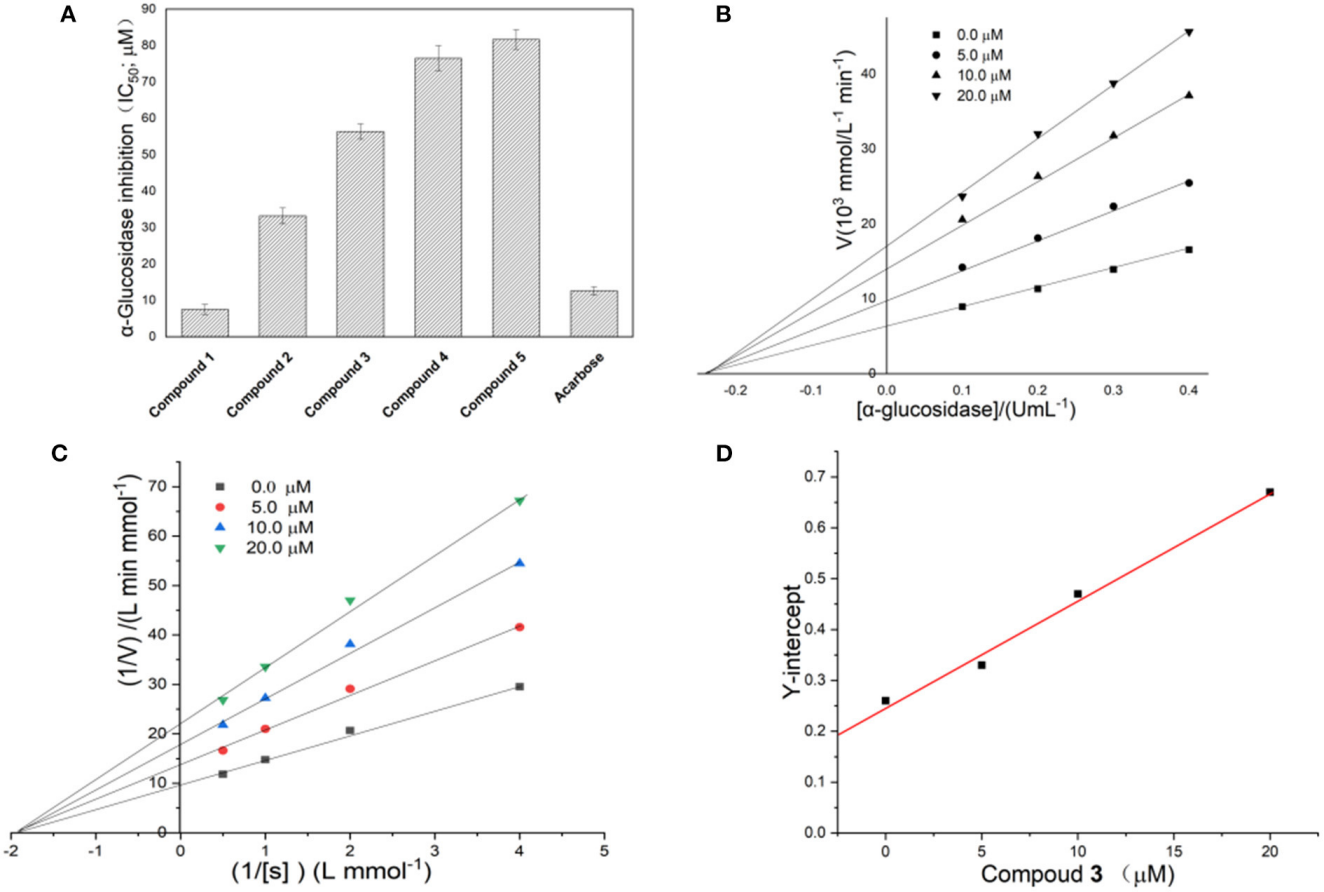
**(A)** Inhibitory activity of compounds **1**–**5** on α-glucosidase; **(B,C)** Linewever-Burk plots of α-glucosidase inhibition. **(D)** Secondary replot of the Y-intercept vs. the concentration of compound **1**.

To determine which ingredients in Fenghuang Dancong tea are the most effective in inhibiting α-glucosidase, we measured the inhibition rates of five compounds. The IC_50_ values of compounds **1**, **2**, **3**, **4**, and **5** were 7.51 ± 1.41 μM, 33.23 ± 2.21 μM, 56.41 ± 2.11 μM, 76.5 ± 3.52 μM, and 81.7 ± 2.72 μM, respectively (Figure 4A). The inhibitory activity of compound **1** was the highest, followed by compounds **2**, **3**, **4**, and **5**. There have been many studies on flavonoids with strong anti-diabetes activity. However, the specific structure-activity relationship of flavonoids on the inhibitory activity of α-glucosidase could not be obtained. In this paper, some correlations can be summarized: the inhibitory activities of compounds **1** and **2** with the parent dihydroflavone were significantly stronger than those of compounds **3**, **4** and **5** with the parent isoflavones. The double bond configuration on the substituent had a certain effect on activity. For example, compound **1** containing *cis* double bonds in the substituent was more active than compound **2** containing *trans* double bonds. The influence of the rigidity and flexibility of substituents on the activity cannot be ignored. Compound **3**, containing a flexible seven-membered ring, was stronger than compounds **4** and **5**, which contained a rigid six-membered ring.

#### Reversibility of the inhibition of α-glucosidase

The reversibility of enzyme inhibition can be determined according to the influence of sample concentration and enzyme concentration on enzyme activity. Figure 4B shows the fitting of straight lines between enzyme concentration and enzyme activity under a series of sample dosages passed through one point, of which the Y-intercept decreased with increasing sample concentration. This implied that **1** was a reversible α-glucosidase inhibitor (16). The reversibility of the inhibition of α-glucosidase by different flavonoids has been studied extensively (17). However, definitive conclusions cannot been drawn about the relationship between the structure of flavonoids and the reversibility of the inhibition of α-glucosidase (18).

#### Inhibitory kinetics of compound 1 on α-glucosidase

The double-reciprocal diagram, based on a Lineweaver–Burk plot, is widely used to investigate inhibitory kinetics of compounds on enzymes. Figure 4C shows the Y-intercept decreased as the concentration of compound **1** increased, while the X-intercept did not change with increasing concentration of compound **1**. This result implied the inhibition of α-glucosidase by compound **1** was non-competitive. Thus, the complex formed by the combination of compound **1** with substrate and α-glucosidase led to the inactivation of the latter. In addition, the number of binding sites between the compound and α-glucosidase can be obtained by a second linear fitting of the concentration of compound **1** and Y-intercept, which is shown in Figure 4D. A linear correlation was observed between the concentration of compound **1** and the Y-intercept, suggesting that compound **1** has one binding site with α-glucosidase. Through further calculation, the binding constant was obtained as 4.58 × 10^4^ L/mol. There were obvious differences in the inhibition mechanism of different types of flavonoids on α-glucosidase. For example, galangin showed mixed inhibition of competitive inhibition and non-competitive inhibition and had one binding site with α-glucosidase (3). Among the four flavonoids isolated from *Sophora flavescens* root, two showed non-competitive inhibition and the other two showed anti-competitive inhibition (17). The flavonoids from *Pueraria lobata* showed non-competitive inhibition (18). The above facts indicate that the mechanisms of action of flavonoids on glucosidase are diverse and complex.

### Glucose consumption activities of compounds 1–5

**Figure 5:**
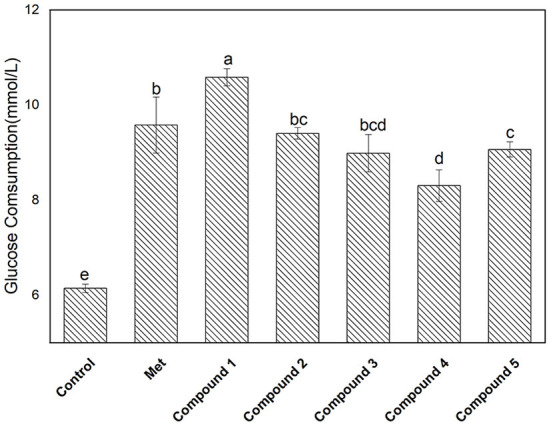
Glucose consumption activity of isolated compounds **1**–**5** with a concentration of 0.18 mg/L. The values with different small letters (a–e) indicate a significant difference between different groups at p < 0.05.

The glucose consumption capacity of cells has important implications for diabetic patients. Here, a HepG2 cell model was used to evaluate the effect of compounds **1**–**5** on glucose consumption, and the results are shown in Figure 5. The glucose consumption values of compounds **1**, **2**, **3**, **4**, and **5** in the HepG2 cell model were 10.58 ± 0.18, 9.40 ± 0.12, 8.98 ± 0.39, 8.30± 0.33, and 9.06 ± 0.15 mmol/L, respectively. The glucose consumption of compound **1** was the highest, followed by compounds **2** > **5** > **3** > **4**. It is well-known that the sugar level in cells is closely related to the amount of insulin secretion or the status of insulin receptors. After the compound was incubated with the cell, the glucose content in the cell decreased, indicating interactions between the compound and the receptor may occur. In addition, compound **1** exhibited outstanding α-glucosidase inhibition and glucose consumption activity. Moreover, the correlation between the two is worthy of further investigation.

## Conclusion

In this paper, bioassay-guided isolation of antidiabetic compounds from an EtOAc extract of Fenghuang Dancong tea was preformed and α-glucosidase activity and their effects on glucose consumption by HepG2 cells were evaluated. From the active fractions of the bioassay-guided isolation, three novel flavones were obtained, namely, amelliaone **F**–**G**. Amelliaone **F** contained a *cis* double bond in the substituent, which led to the highest inhibition of α-glucosidase, with an IC_50_ value of 7.51 μM, and the highest glucose consumption by HepG2 cells, of 10.58 ± 0.18 mmol/L. These results indicate that the EtOAc extract of Fenghuang Dancong tea could be a potential source of antidiabetic compounds.

## Data availability statement

The original contributions presented in the study are included in the article/Supplementary material, further inquiries can be directed to the corresponding author.

## Author contributions

Conceptualization, data curation, writing—original draft, and funding acquisition: HZ. Methodology: HZ, JLia, JO, and JLin. Investigation: HZ and JL. Formal analysis: JLia, JO, JLin, and YL. Writing—review and editing: HZ, JZ, SO, and FL. All authors contributed to the article and approved the submitted version.

## Funding

This work was financially supported by the NSFC of China (Nos. 31101323 and 31671957) and the high-performance computing platform of Jinan University.

## Conflict of interest

The authors declare that the research was conducted in the absence of any commercial or financial relationships that could be construed as a potential conflict of interest.

## Publisher's note

All claims expressed in this article are solely those of the authors and do not necessarily represent those of their affiliated organizations, or those of the publisher, the editors and the reviewers. Any product that may be evaluated in this article, or claim that may be made by its manufacturer, is not guaranteed or endorsed by the publisher.
