# Supplementary material for: Bioassay-guided isolation of Fenghuang Dancong tea constituents with α-glucosidase inhibition activities
Source: Front Nutr. 2022 Nov 10;9:1050614. doi: 10.3389/fnut.2022.1050614 (PMC9686339; doi:10.3389/fnut.2022.1050614)
Supplement: Supplementary file 1 [file Data_Sheet_1.pdf]

Supplementary Material

Supplementary Figures

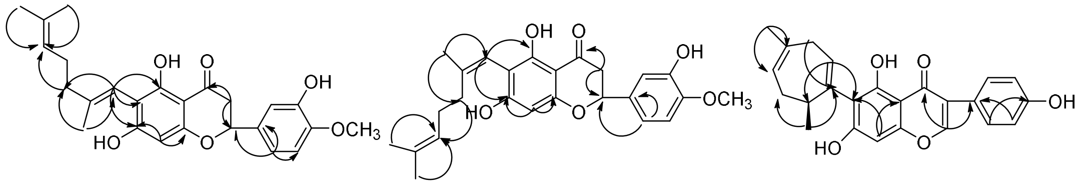

1 2 3

Supplementary Figure 1. HMBC of compounds **1**–**3** isolated from Fenghuang tea.

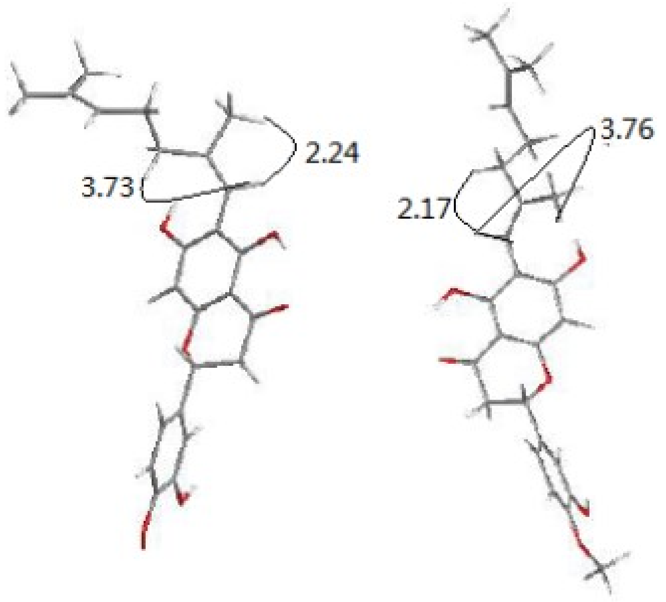

Supplementary Figure 2. Two isoforms of compound **1** and **2** in minimization energy.

Formula	Intensity	Threshold	Expected m/z	Found at m/z	Error (ppm)	Expected RT (min)	Found RT (min)	RT Delta (min)	Isotope Diff (%)
C25H28 O6	803745	50	425.1959	425.1961	0.6	0.00	0.07	0.07	6.3%

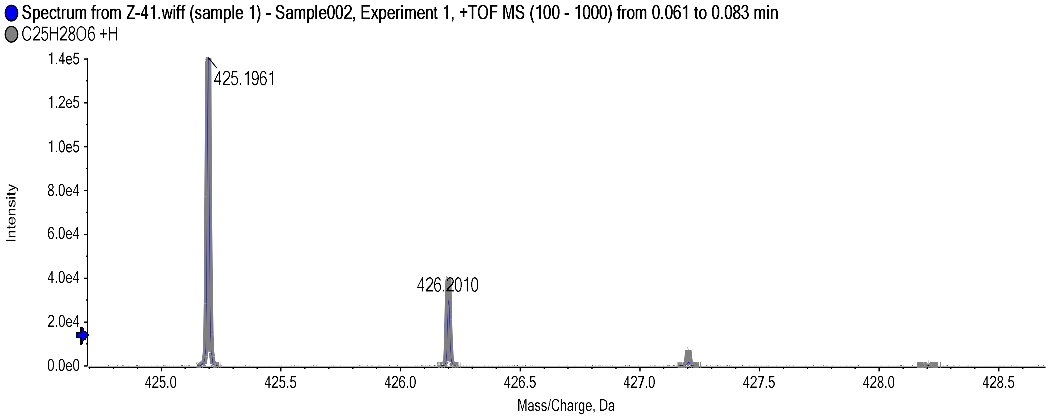

Supplementary Figure 3. HR-ESI-MS data of compound **1**

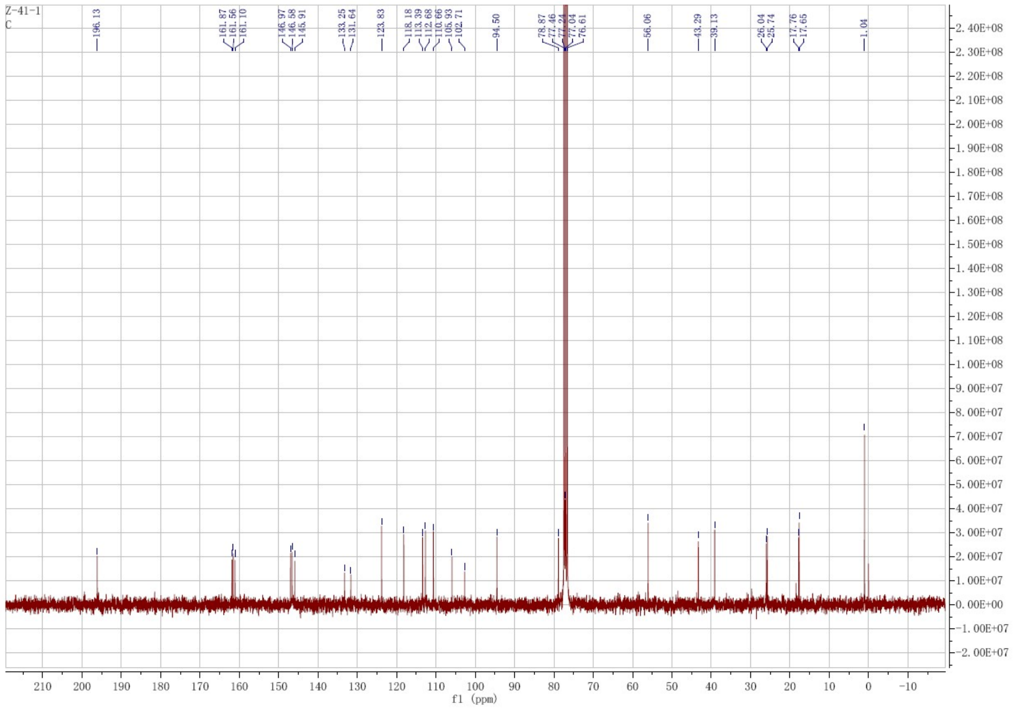

Supplementary Figure 4. ^{13}C NMR spectrum of compound 1 (in CD_3Cl, 100 MHz)

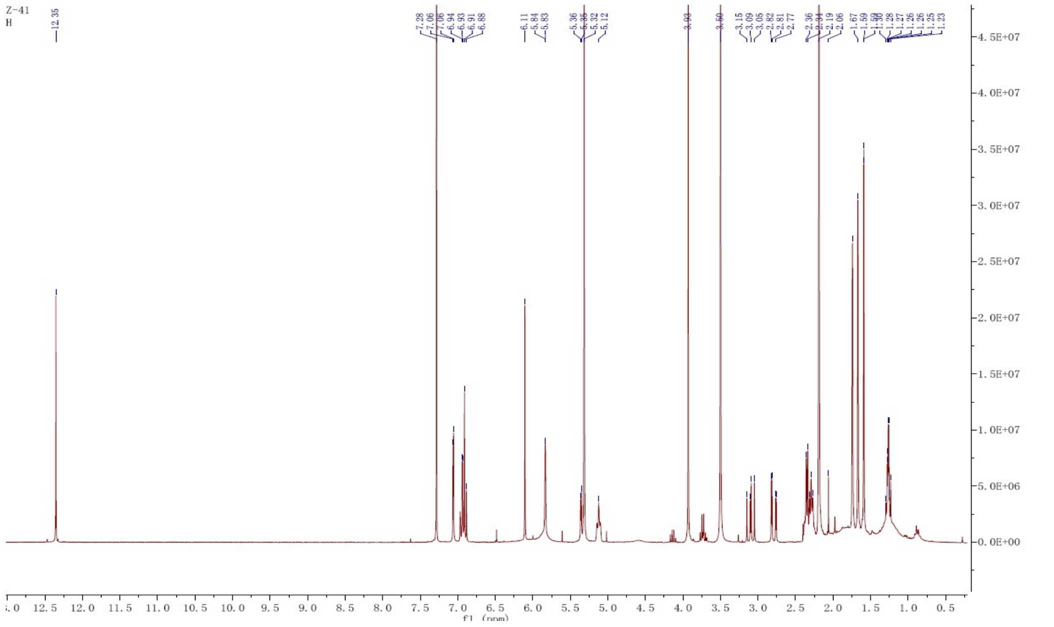

Supplementary Fgure 5. ^{1}H NMR spectrum of compound 1 (in CD_3Cl, 400 MHz)

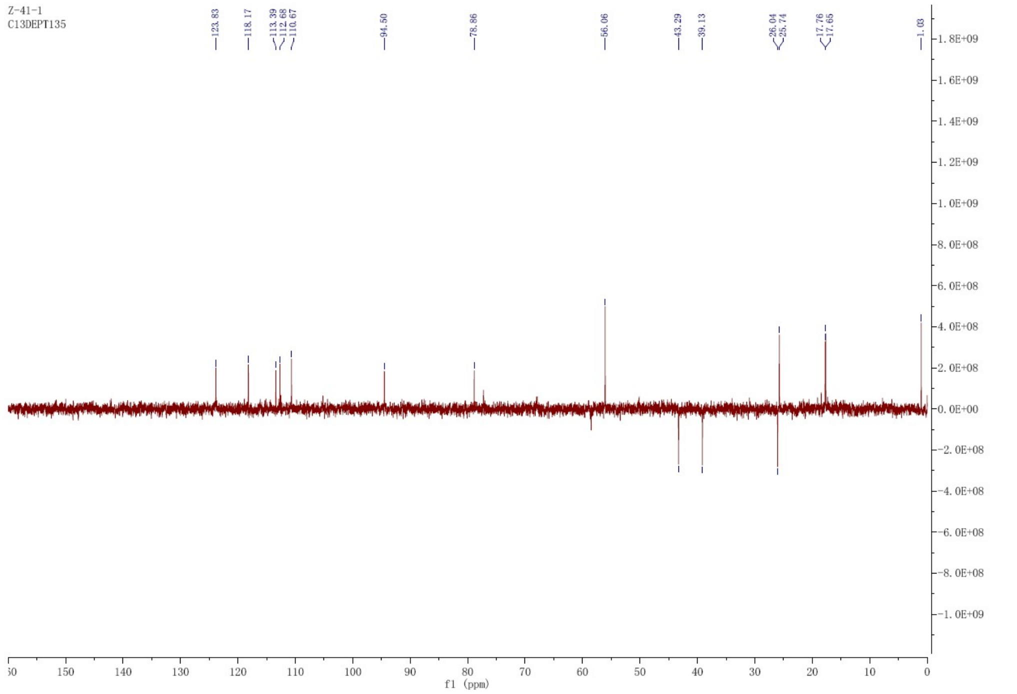

Supplementary Fgure 6. DEPT-135 spectrum of compound **1** (in CD_3Cl)

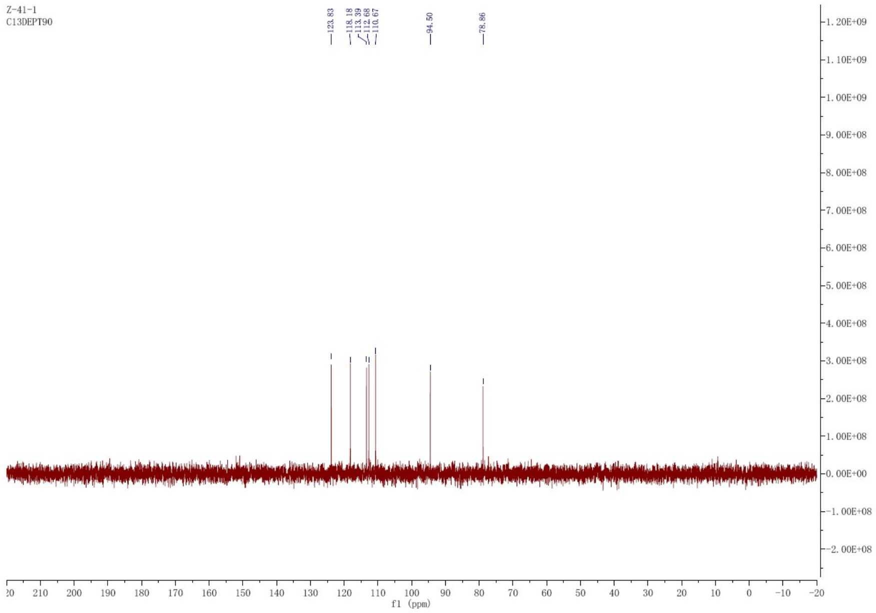

Supplementary Fgure 7. DEPT-90 spectrum of compound **1** (in CD_3Cl)

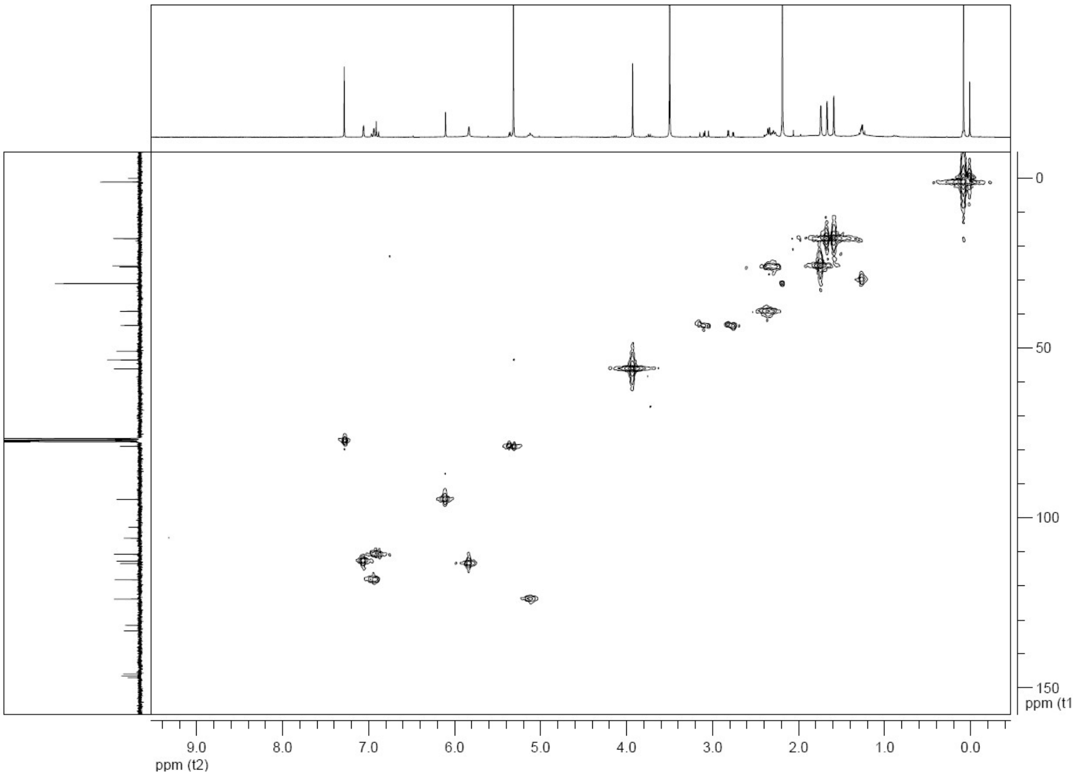

Supplementary Fgure 8. HSQC spectrum of compound **1** (in CD_3Cl)

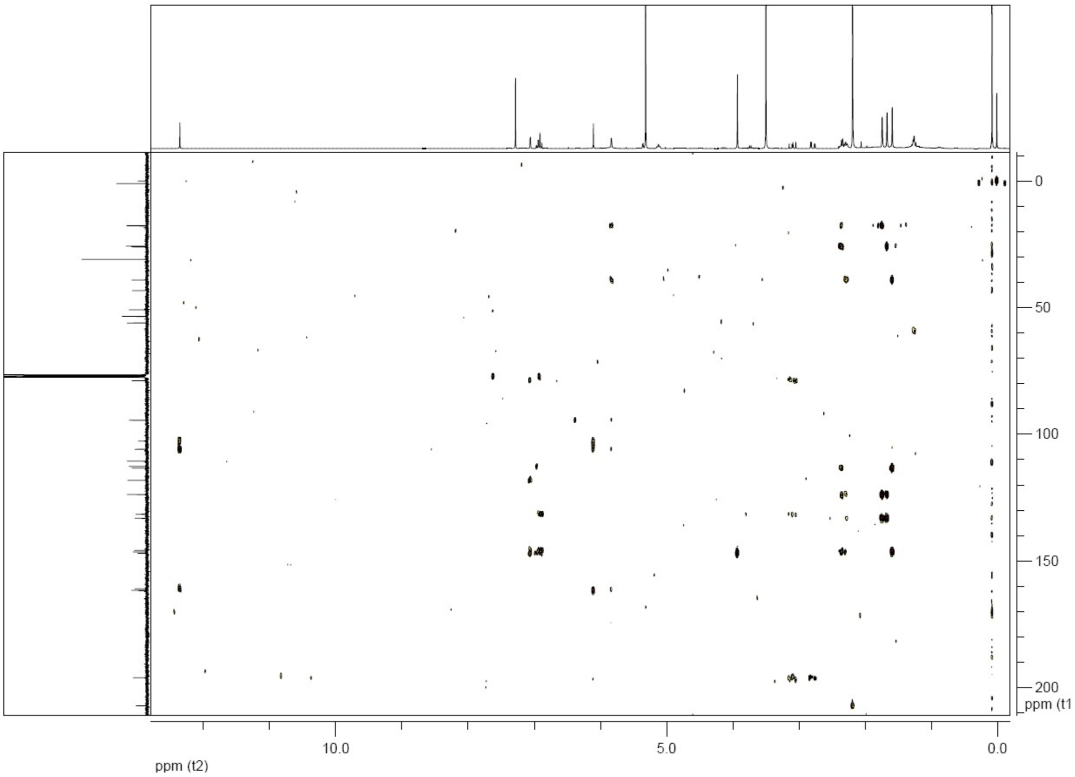

Supplementary Fgure 9. HMBC spectrum of compound **1** (in CD_3Cl)

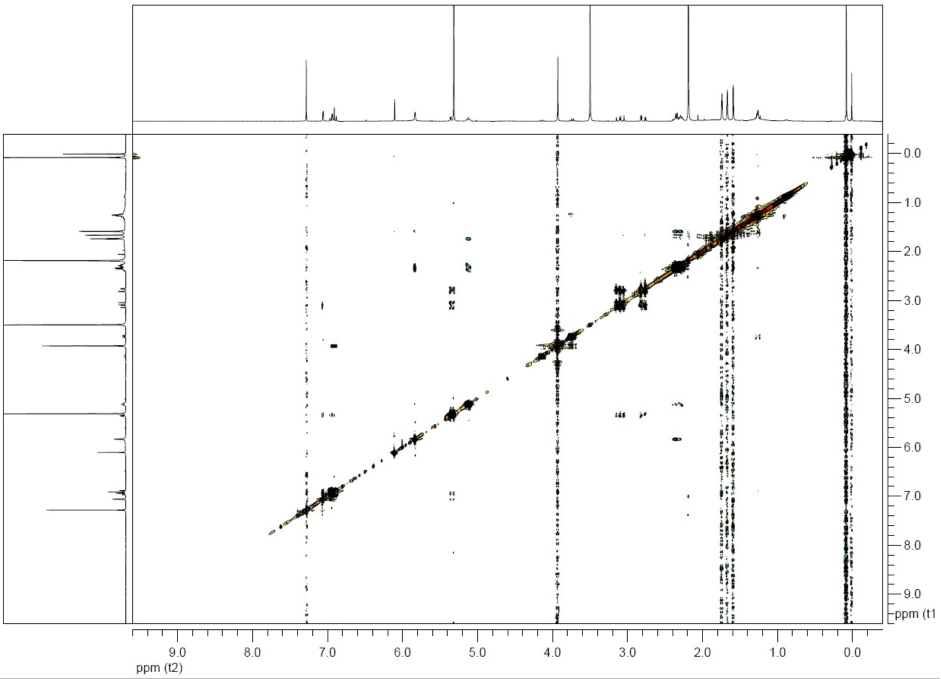

Supplementary Fgure 10. ^1H-^1H ROESY spectrum of compound **1** (in CD_3Cl, 400 MHz)

Formula	Intensity	Threshold	Expected m/z	Found at m/z	Error (ppm)	Expected RT (min)	Found RT (min)	RT Delta (min)	Isotope Diff (%)	Purity (%)
C25H28O6	1154769	50	425.1959	425.1963	1.0	0.00	0.08	0.08	12.8%	0.0%

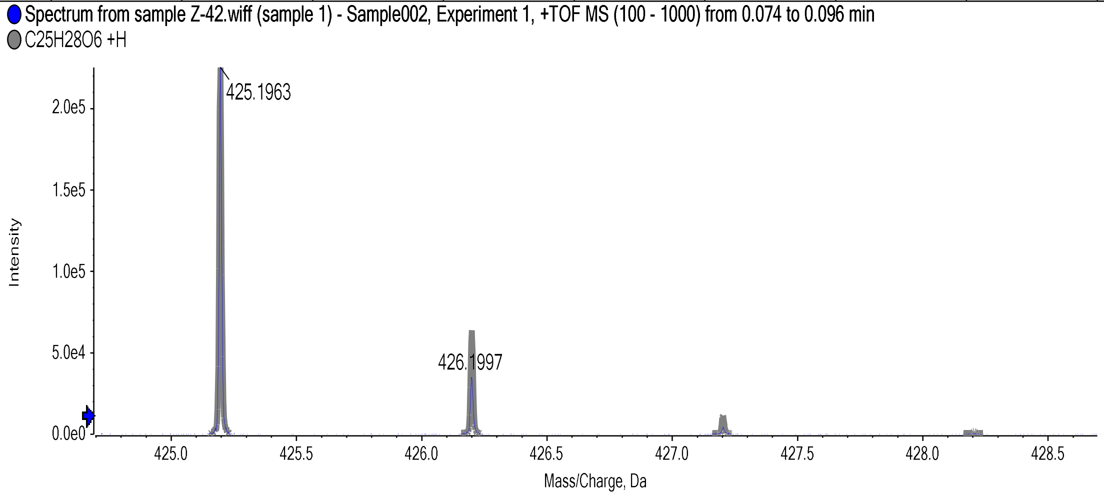

Supplementary Figure 11. HR-ESI-MS data of compound **2**

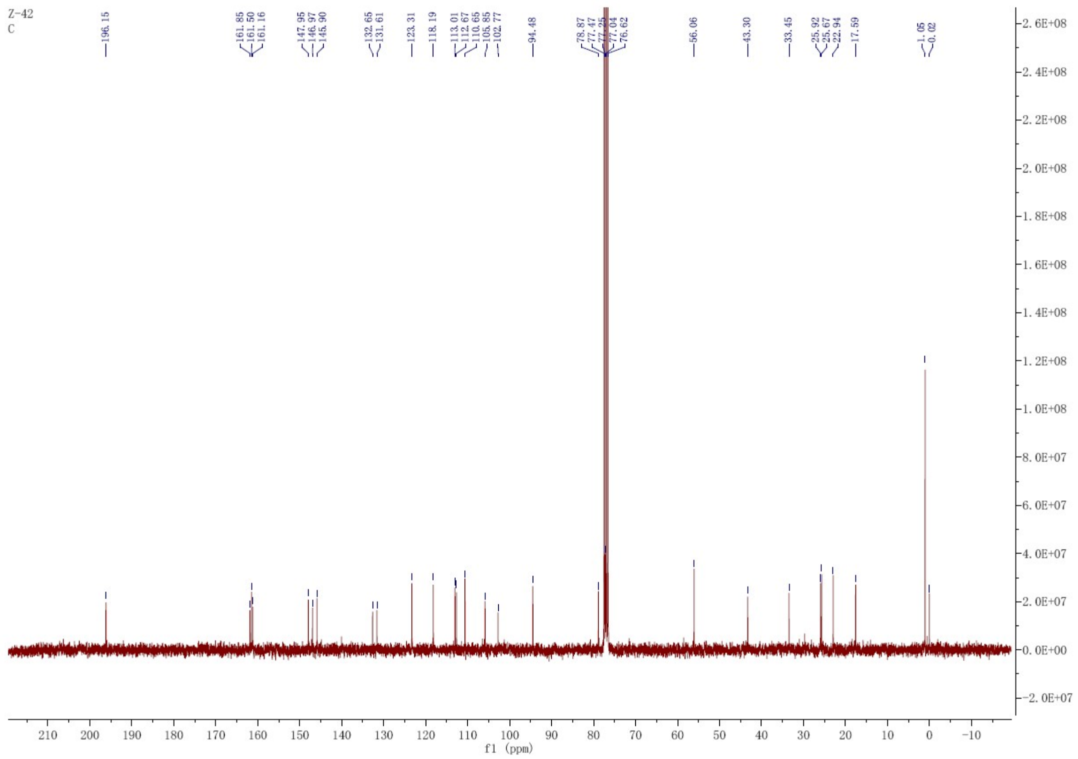

Supplementary Figure 12. ^{13}C NMR spectrum of compound **2** (in CD3Cl, 100 MHz)

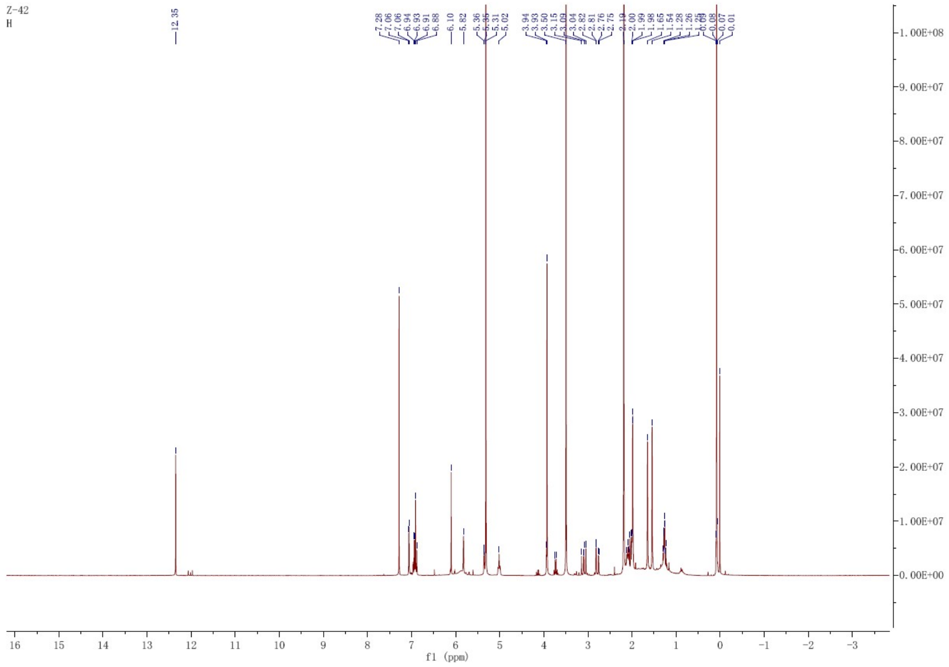

Supplementary Figure 13. ^{1}H NMR spectrum of compound **2** (in CD_3Cl, 400 MHz)

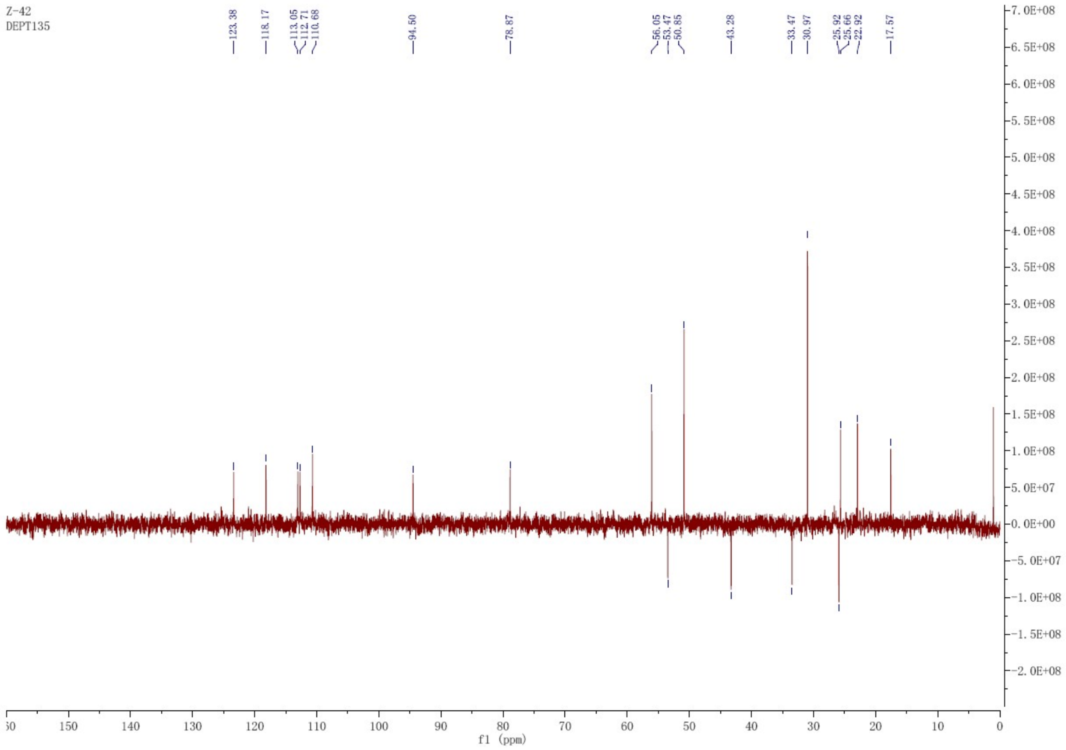

Supplementary Figure 14. DEPT-135 spectrum of compound **2** (in CD_3Cl)

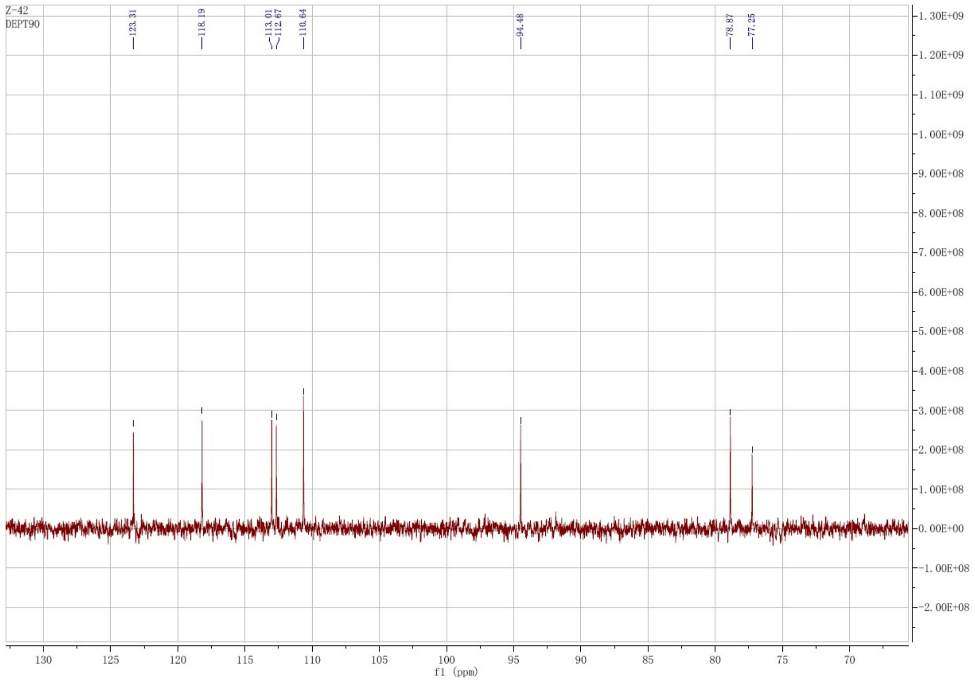

Supplementary Figure 15. DEPT-90 spectrum of compound **2** (in CD3Cl)

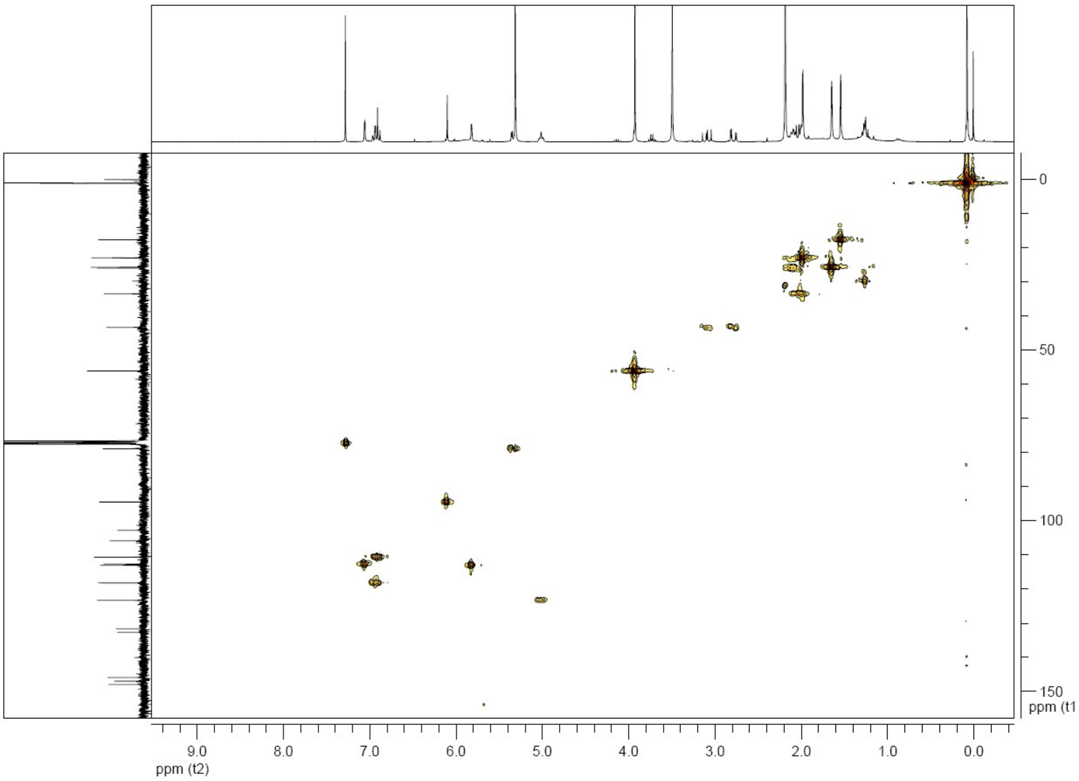

Supplementary Figure 16. HSQC spectrum of compound **2** (in CD3Cl)

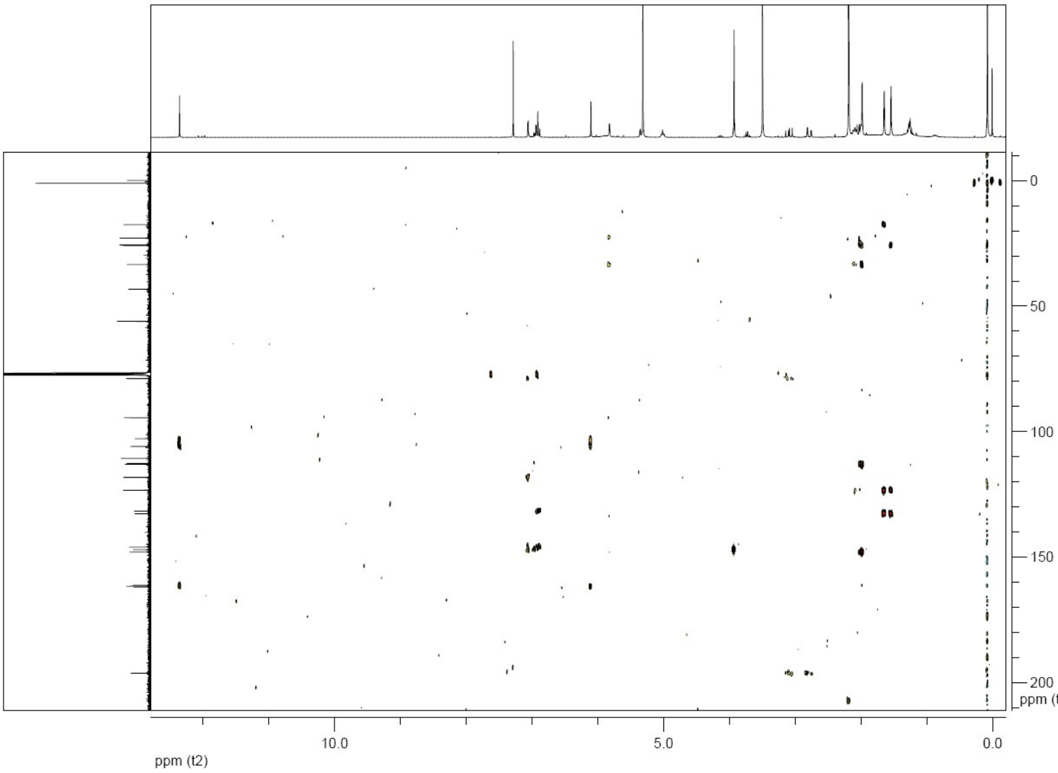

Supplementary Figure 17. HMBC spectrum of compound **2** (in CD_3Cl)

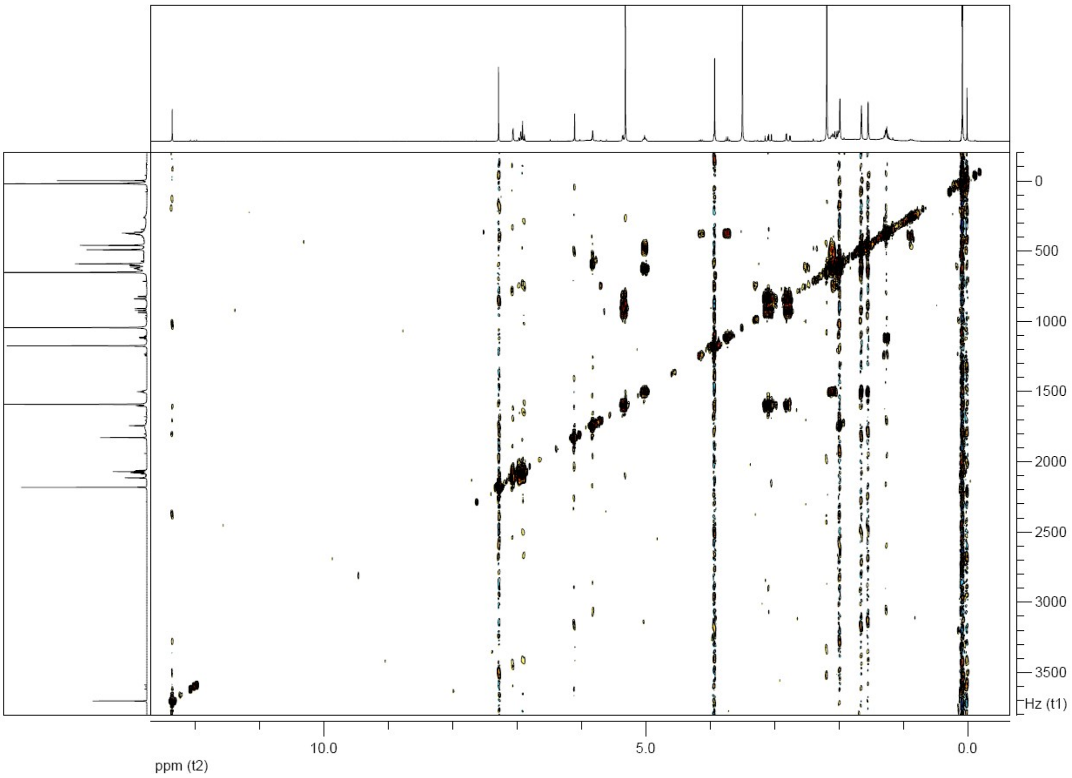

Supplementary Figure 18. ^1H-^1H ROESY spectrum of compound **2** (in CD_3Cl, 400 MHz)

Formula	Intensity	Threshold	Expected m/z	Found at m/z	Error (ppm)	Expected RT (min)	Found RT (min)	RT Delta (min)	Isotope Diff (%)
C24H22O5	2944202	50	391.1540	391.1542	0.6	0.00	0.09	0.09	5.2%

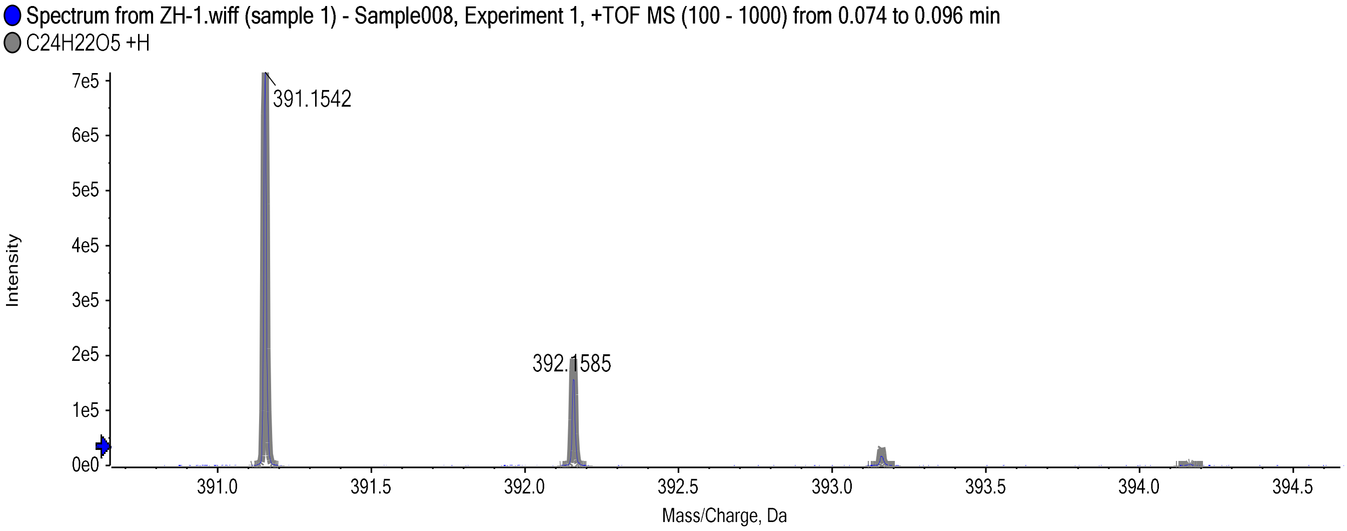

Supplementary Figure 19. HR-ESI-MS data of compound **3**

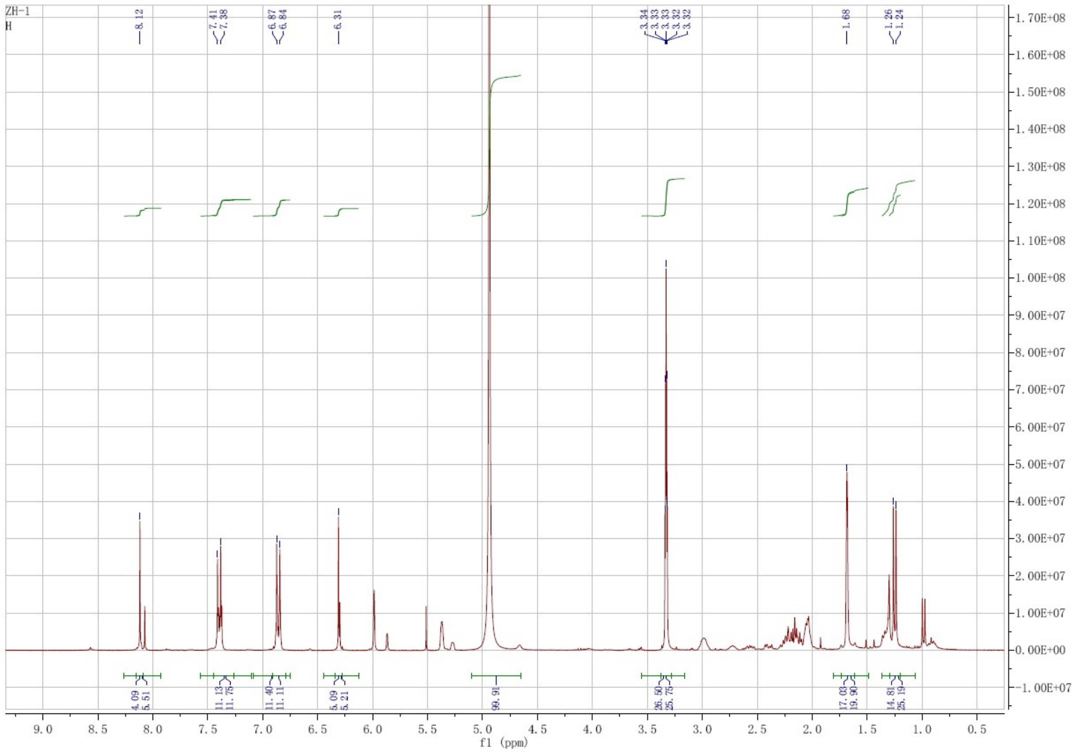

Supplementary Figure 20. ^{1}H NMR spectrum of compound **3** (in CD_3OD, 400 MHz)

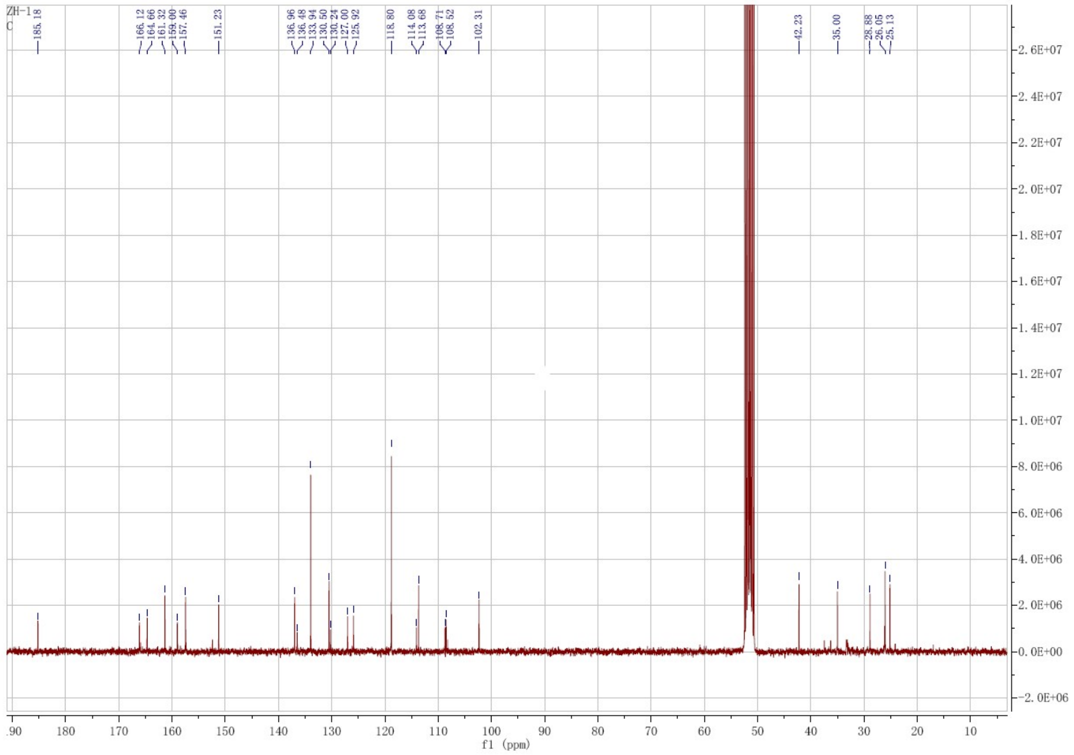

Supplementary Figure 21. ^{13}C NMR spectrum of compound **3** (in CD_3OD, 100 MHz)

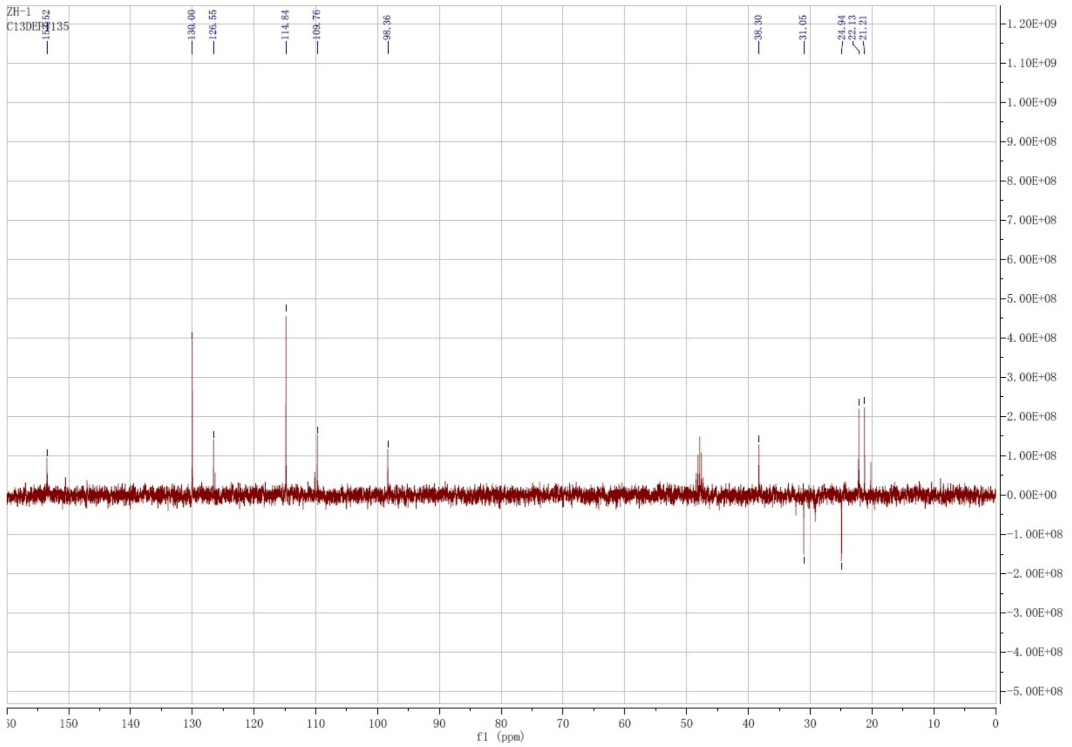

Supplementary Figure 22. DEPT-135 spectrum of compound **3** (in CD_3OD)

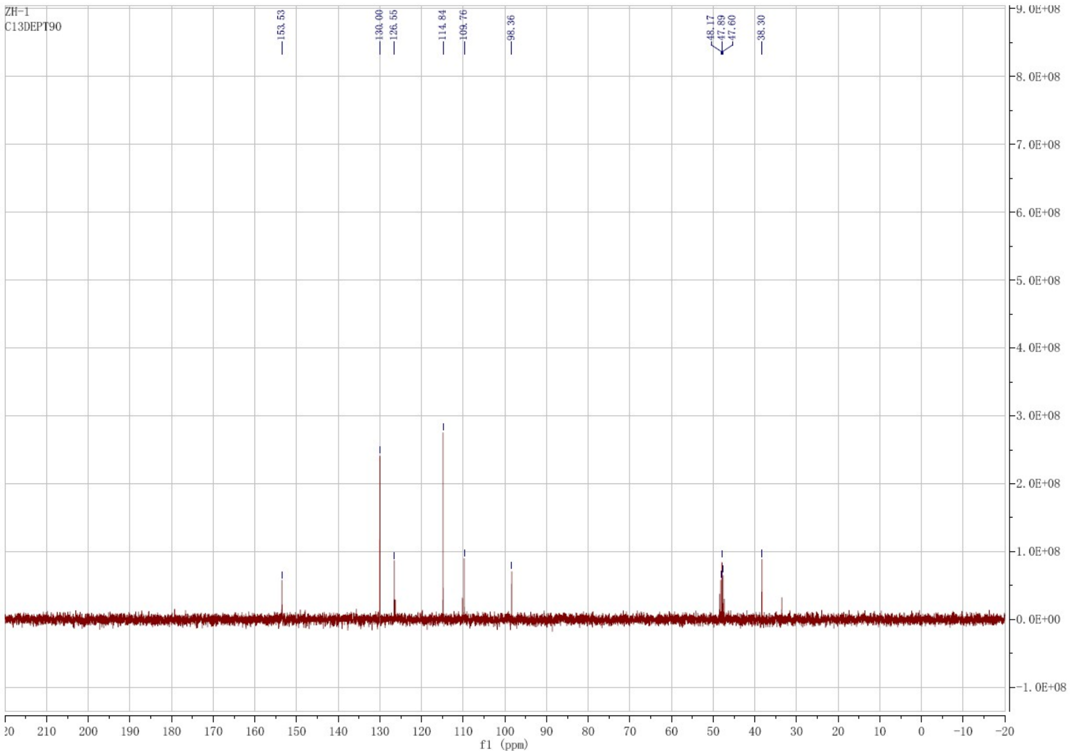

Supplementary Figure 23. DEPT-90 spectrum of compound **3** (in CD_3OD)

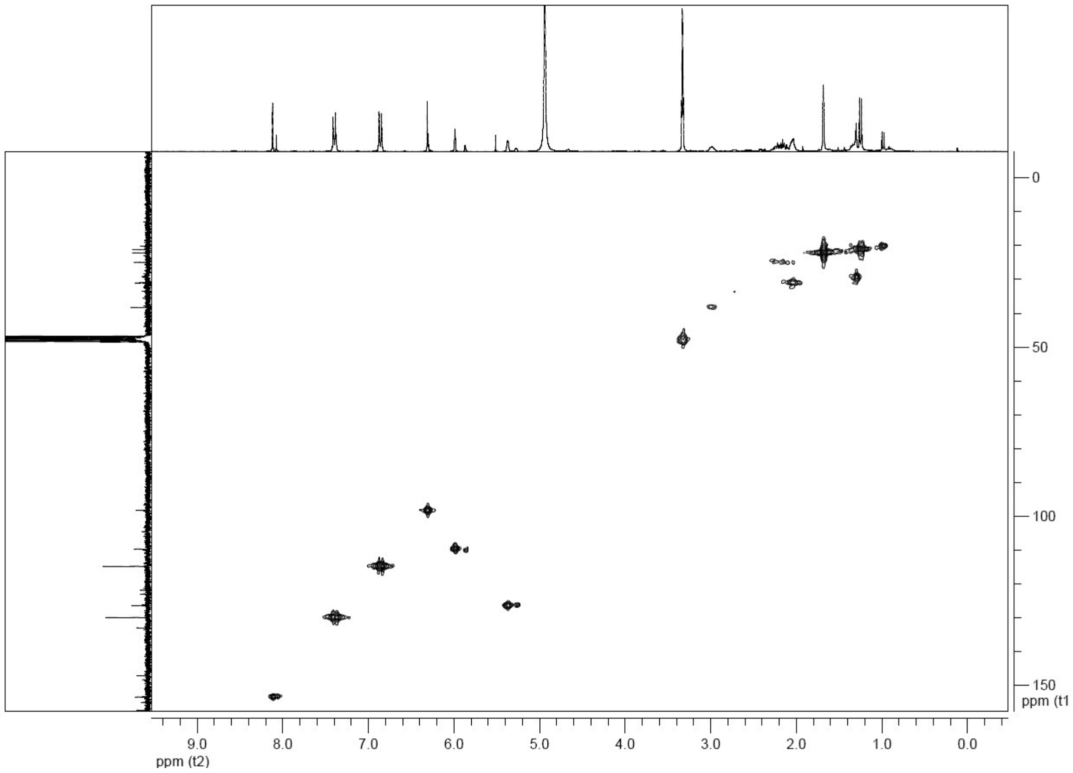

Supplementary Figure 24. HSQC spectrum of compound **3** (in CD_3OD)

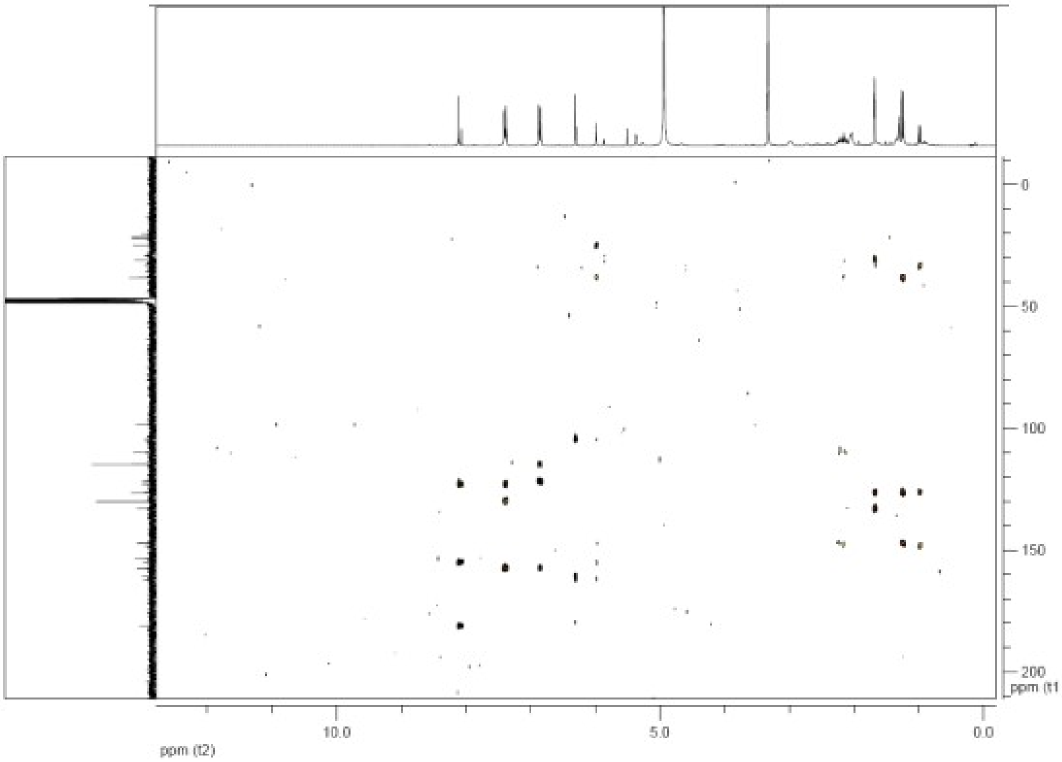

Supplementary Figure 25. HMBC spectrum of compound **3** (in CD_3OD)

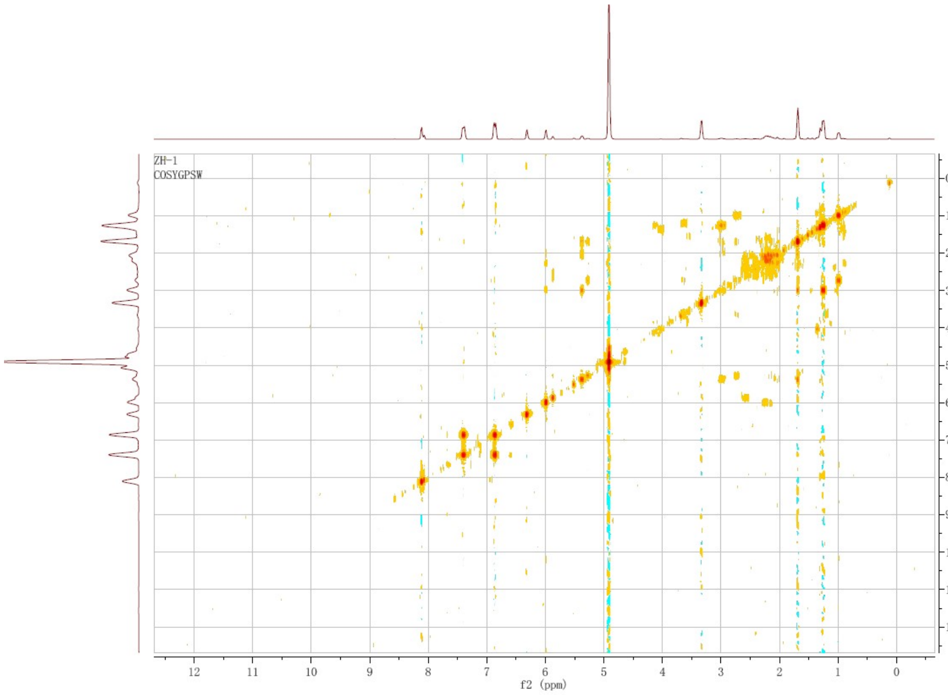

Supplementary Figure 26. ^1H-^1H COSY spectrum of compound **3** (in CD_3OD)

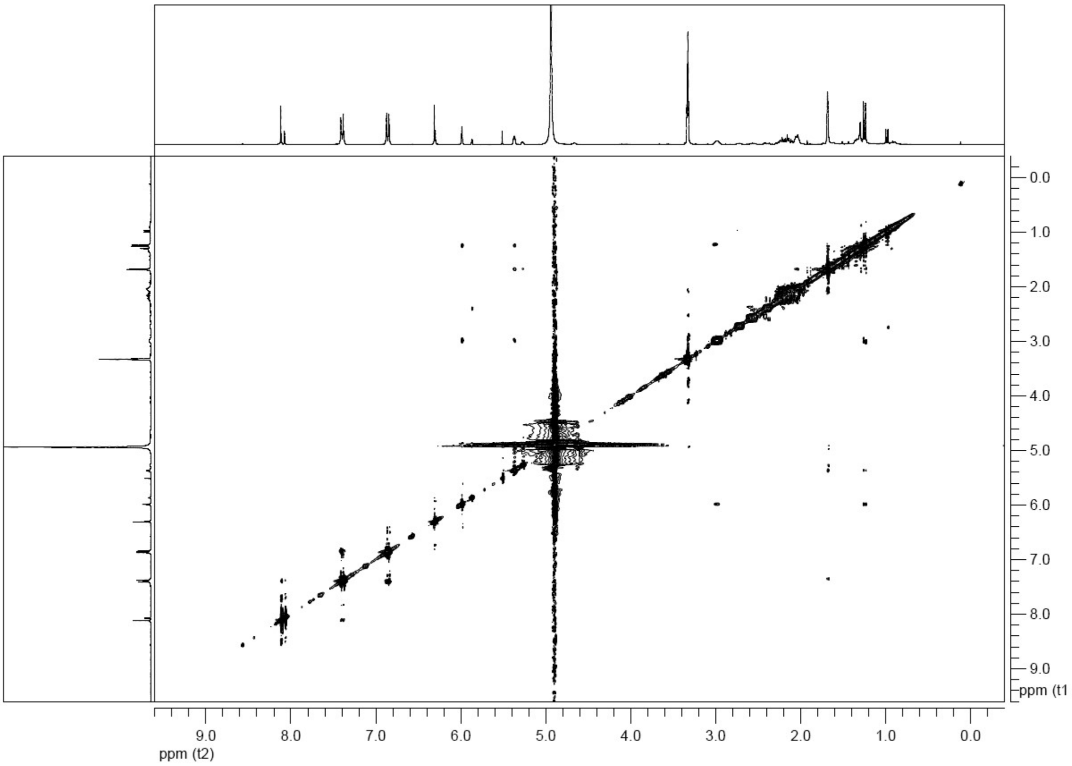

Supplementary Figure 27. ^1H-^1H ROESY spectrum of compound **3** (in CD_3OD)

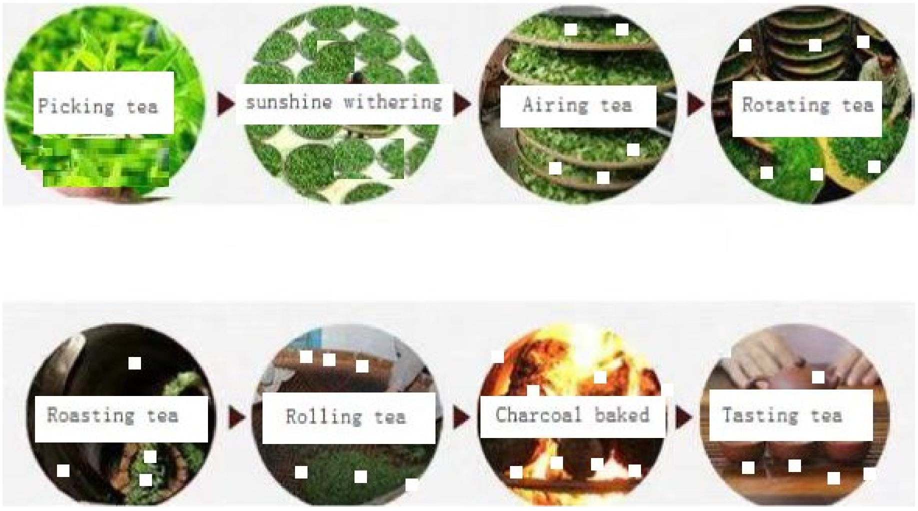

Supplementary Figure 28. Schematic diagram of tea Processing
